# Children's Country Holidays Fund

**Published:** 1914-07-18

**Authors:** 

**Affiliations:** Chairman of the London County Council. Chairman's Room, County Hall, Spring Gardens, London, S.W.


*To the Editor of* The Hospital.

### Children's Country Holidays Fund.

*To the Editor of* The Hospital.

Sir,—As chairman of the body which is the local education authority for London, and which is concerned with the welfare of some 750,000 children, I am writing to you to enlist your sympathy in a cause which must appeal strongly to the citizens of London at this time of year.

I need not remind your readers how the London County Council is carrying on day by day a great work for the children of London through its system of education, not only training their characters and minds, but also caring for their physical well-being by means of physical exercises, organised games, playground classes, school journeys, open-air schools, and medical supervision. I need not recall to them what opportunities for healthy recreation are provided for the children in the parks and open spaces that the Council controls.

In spite, however, of all that the Council can do, the fact remains that, when holiday time comes round, the children naturally crave for a change from their ordinary surroundings, and there are many who look with envying eyes on their more fortunate neighbours who are able to go away into country air and country scenes, where they can roam in fields and woods and see with their own eyes the wonders of flower and bird and butterfly. Would it not be a good plan for everyone who takes his own child away for a holiday, or goes for a holiday alone, to send a contribution to the Children's Country Holidays Fund at the same time? The amount could be such as would suit all pockets, for ten shillings gives a fortnight's holiday to one child, and four half-crowns would therefore provide the fortnight, while a £5-note would give ten whole fortnights to lucky little people. Those who did this would, I have no doubt, double the enjoyment of their own holiday by giving joy to another.

I understand that this year money is not coming in to the Children's Country Holidays Fund so well as in previous years, and the Society is at present nearly £1,000 short of subscriptions. I earnestly hope that all who are able to spare something towards brightening the holiday time of our London children will send a contribution at once to the Treasurer, the Earl of Arran, 18 Buckingham Street, Strand, W.C.—I am, Sir, your obedient servant,

Peel,
Chairman of the London County Council.

Chairman's Room, County Hall,
Spring Gardens, London, S.W.
July 10, 1914.

